# Supplementary material for: Multi-Task Deep Learning Model for Classification of Dental Implant Brand and Treatment Stage Using Dental Panoramic Radiograph Images
Source: Biomolecules. 2021 May 30;11(6):815. doi: 10.3390/biom11060815 (PMC8226505; doi:10.3390/biom11060815)

Figure S1. Mean ROC curves of each CNN models for 12 types of dental implant classification and treatment stage.

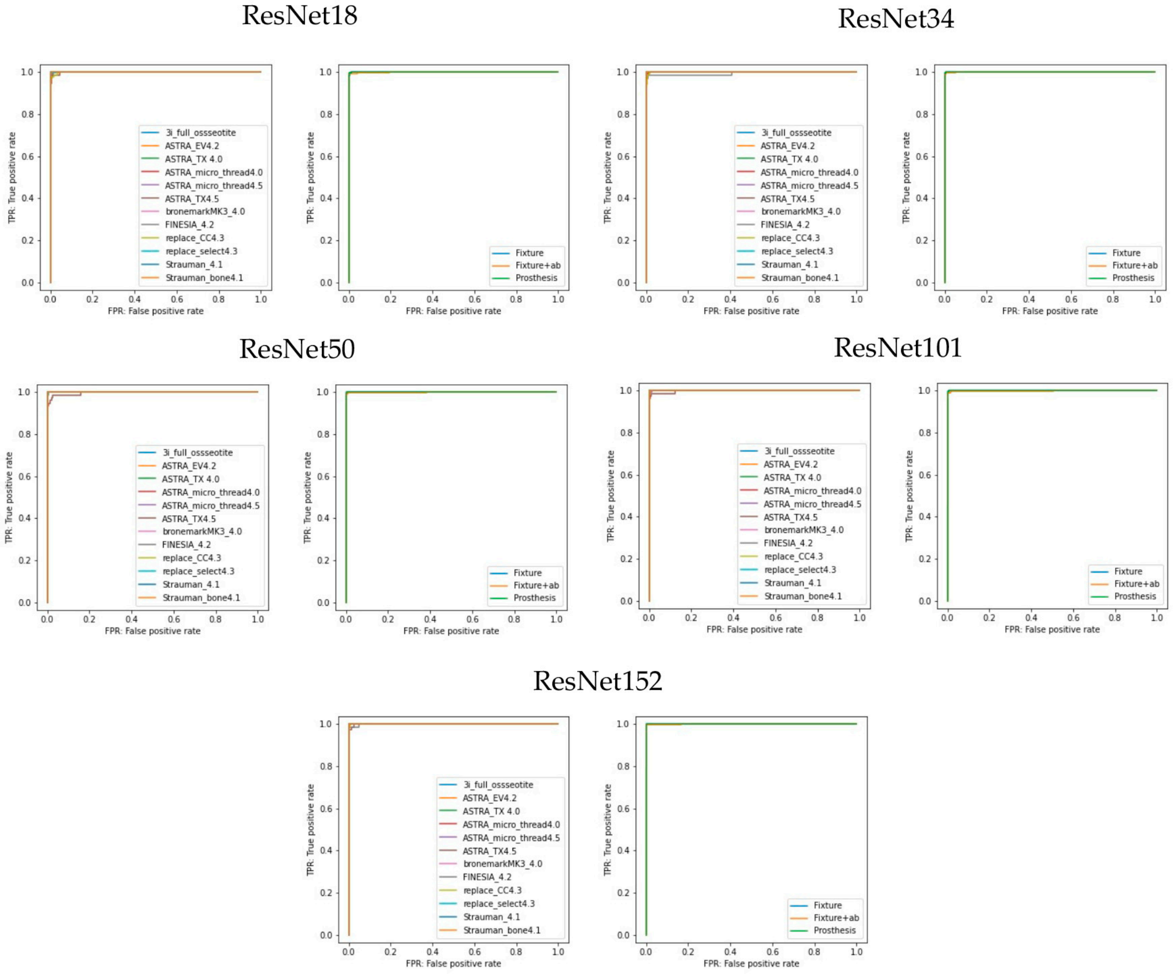

Supplement: Supplementary file 1 [file biomolecules-11-00815-s001.zip › biomolecules-1211797-supplementary.pdf]
